# Pre-Columbian origins of Native American dog breeds, with only limited replacement by European dogs, confirmed by mtDNA analysis

**DOI:** 10.1098/rspb.2013.1142

**Published:** 2013-09-07

**Authors:** Barbara van Asch, Ai-bing Zhang, Mattias C. R. Oskarsson, Cornelya F. C. Klütsch, António Amorim, Peter Savolainen

**Affiliations:** 1 IPATIMUP, Rua Dr Roberto Frias s / n, 4200-465 Porto, Portugal; 2 Faculdade de Ciências da Universidade do Porto, Rua do Campo Alegre s/n, 4169-007 Porto, Portugal; 3 Science for Life Laboratory, School of Biotechnology, Division of Gene Technology, KTH-Royal Institute of Technology, SE-171 65 Solna, Sweden; 4 Bethany College, Lindsborg, KS 67456, USA; 5 College of Life Sciences, Capital Normal University, Beijing 100048, People's Republic of China

**Keywords:** dog, mitochondrial DNA, mtDNA, haplotype, America, pre-Columbian

## Abstract

Dogs were present in pre-Columbian America, presumably brought by early human migrants from Asia. Studies of free-ranging village/street dogs have indicated almost total replacement of these original dogs by European dogs, but the extent to which Arctic, North and South American breeds are descendants of the original population remains to be assessed. Using a comprehensive phylogeographic analysis, we traced the origin of the mitochondrial DNA lineages for Inuit, Eskimo and Greenland dogs, Alaskan Malamute, Chihuahua, xoloitzcuintli and perro sín pelo del Peru, by comparing to extensive samples of East Asian (*n* = 984) and European dogs (*n* = 639), and previously published pre-Columbian sequences. Evidence for a pre-Columbian origin was found for all these breeds, except Alaskan Malamute for which results were ambigous. No European influence was indicated for the Arctic breeds Inuit, Eskimo and Greenland dog, and North/South American breeds had at most 30% European female lineages, suggesting marginal replacement by European dogs. Genetic continuity through time was shown by the sharing of a unique haplotype between the Mexican breed Chihuahua and ancient Mexican samples. We also analysed free-ranging dogs, confirming limited pre-Columbian ancestry overall, but also identifying pockets of remaining populations with high proportion of indigenous ancestry, and we provide the first DNA-based evidence that the Carolina dog, a free-ranging population in the USA, may have an ancient Asian origin.

## 1. Introduction

The dog is now well established as the most ancient domestic animal and is unique as the only domesticate present in human societies on every continent in ancient times [1]. Previous studies of mitochondrial (mt) DNA have indicated southern East Asia as the geographical centre for the origin of dogs [2–5]. This has been largely supported by analyses of Y-chromosomal DNA [5,6], while a recent single nucleotide polymorphism (SNP)-based study argued that Middle Eastern wolves primarily contributed to the extant dog gene pool [7]. Regardless of the exact geographical origin of domestic dogs, it seems clear that American dogs originate from the Old World since analysis of ancient samples from several pre-Columbian archaeological sites were shown to have the same mtDNA haplogroups as Old World dogs [8].

A remaining question is if today's American dogs trace their origin to the dogs originally introduced from Asia via the Bering Straits in pre-Columbian time, or if this ancestry has been diluted or even completely erased by European dogs brought across the Atlantic following the arrival of Europeans in America. Several studies of mtDNA have attempted to elucidate this question, but did not include comprehensive samples across American breeds and/or of reference samples from the Old World, necessary for a comprehensive analysis [8–11]. Importantly, with the detailed knowledge of the worldwide dog mtDNA diversity presented recently [4], an opportunity has opened up to identify whether mtDNA haplotypes carried by American dogs originate from East Asia in ancient times or from Europe in the post-Columbian era.

Archaeological data and historic records have provided abundant evidence that dogs in pre-Columbian times were part of native cultures in the American continent long before the dawn of transoceanic travel in the fifteenth century [12]. Dogs were used for a large number of different purposes, for example, for hunting, sledging, freighting, protection and company, for religious and medicinal purposes and as a food resource. The earliest archaeological evidence for presence of dogs in the Americas has been dated to 10 000–8500 years ago, the dog thus being the sole domesticate in America during several thousand years [13,14]. According to current hypotheses, the Americas started to be colonized by south Siberian peoples at least 15 000 years ago immediately after deglaciation of the Pacific coastal corridor [15]. Thus, pre-Columbian dogs must have been brought along by Paleo-Indians of Asian origin in their expansions throughout the American continent, although not necessarily in connection with the first waves of humans.

A small number of extant breeds in the American continent, for example, the Mexican Chihuahua, the xoloitzcuintli (Mexican hairless dog) and the Peruvian perro sín pelo (Peruvian hairless dog) have been claimed to descend from pre-Columbian populations. Archaeological findings in South America are abundant in evidence of dogs increasingly entwined in human societies [16]. In the Arctic region, the presence of well-adapted dogs and their use by Native American peoples prior to 1492 is also well recorded [17]. Many of those dogs, such as the hare Indian dog and the Tahltan bear dog have disappeared as the aboriginal hunting methods declined. Modern Arctic breeds thought to be indigenous include the Inuit sled dog, the Canadian Eskimo dog and the Greenland dog (all three thought to share the same origin) and the Alaskan Malamute. Some modern American breeds such as the dogo Argentino, the fox Paulistinha, the fila brasileiro and the cimarrón Uruguayo have historical origins in European dogs and are not putatively indigenous. Similarly, the Alaskan husky and the American Eskimo dog have a known origin from Siberian spitzes and European dogs.

Besides breed dogs, free-ranging dogs are abundantly found across the American continent. These dogs commonly have heterogeneous morphologies suggesting an origin predominantly from a mix of European breeds. However, in remote areas of southeastern USA (South Carolina and Georgia) there is a group of free-ranging dogs (called the Carolina dog) which morphologically resemble the Australian dingo and South Asian pariah dogs. Based on this resemblance to ‘primitive’ dogs, an origin from indigenous pre-Columbian dogs rather than from run-away breed dogs of European origin has been suggested [18].

The strong impact of the introduction of domestic species by European settlers and explorers, and its deleterious effects on Native American cultures, is indisputable. The replacement or dilution of Native American dog populations with European dogs is thought to have been severe, with consequent loss of historic Native breeds [19]. However, the extent of this replacement or admixture remains largely undisclosed. Did the ancient migrants leave descendants in the modern American gene pool or were they completely erased by European dogs brought across the Atlantic in the post-Columbian era, and are extant dog populations direct descendents of the ancient populations in the same geographical region?

A very recent study of mtDNA in pre-Columbian dogs from Alaska and Greenland [9] showed that the ancient population and the modern Inuit sled dog population in Greenland carry predominantly the unique haplotype A31, strongly indicating local ancestry for this group of Arctic dogs. By contrast, a study of mtDNA among village dogs and street dogs from across America indicated that the maternal lineages of the indigenous American dog population have been almost completely replaced with European dogs [10]. However, this study did not include American breed dogs. Furthermore, the results were based on comparisons with a restricted source of information, that is, with the haplotypes found among 19 ancient American dog samples analysed in one previous study [8].

Another approach, possibly allowing for a more comprehensive picture of the geographical ancestry of mtDNA lineages among American dogs, is to compare extant American dogs with European and East Asian dogs, assuming that today's populations are good approximations for the ancient ones. The possibility of tracing the geographical origin of mtDNA lineages has opened up with the comprehensive picture of the worldwide dog mtDNA diversity presented recently [4]. This study showed distinct differences between the European and East Asian mtDNA gene pools, offering the possibility to trace whether mtDNA haplotypes among American dogs may have originated from Europe or East Asia. All populations across the Old World share a common gene pool of three major phylogenetic groups called clades A, B and C. In East Asia, the full diversity of these genetic groups is represented, but European dogs (together with all other populations west of the Himalayas and the Urals) have a limited diversity. Out of totally 206 mtDNA haplotypes identified globally [3], there are 15 haplotypes which are represented in virtually every dog population across the Old World, called universal types (UTs). In Europe, 76.7% of the dogs carry one of these 15 haplotypes and totally 92.6% carry a haplotype which is a UT or differs by a single substitution from one of the UTs. In addition, haplogroup D (which is absent in East Asia) has been found in Europe, in 5.9% of the dogs. Thus, totally 98.5% of European dogs carry an mtDNA which is a UT, differs by one substitution from a UT or belongs to haplogroup D. Consequently, if an American dog carries a haplotype which does not belong to this group of haplotypes, it is unlikely that its mtDNA lineage derives from Europe. In East Asia and Siberia, only 54.9% and 50% of the dogs, respectively, carry a UT; instead, there is a large number of unique haplotypes distinct from those in Europe. Therefore, it may be assumed that approximately 50% of the dogs that entered America via Bering Strait in pre-Columbian time carried a haplotype which was not a UT or belonged to haplogroup D and is therefore potentially informative for excluding a European origin.

Importantly, the high frequency of the 15 UTs and haplotypes differing by a single substitution from the UTs is a universal phenomenon west of the Himalayas, with for example 94.5% of dogs in southwest Asia and 93.1% in Africa, carrying such haplotypes [3,4]. Therefore, the high proportion of these haplotypes in today's European population most probably reflects the genetic make-up across western Eurasia in ancient times, and thus also in Europe at the time of European colonization of America. The assumption that today's European population can be used to represent the situation 500 years ago therefore seems justified, offering a firm basis for the analyses in this study.

In this study, the origins of American dogs were addressed by comparison of mtDNA sequences from American, European and East Asian dogs. This dataset includes new samples for several American breeds and free-ranging dog populations and an extensive collection of European dogs. Together with previous data for extant dogs across the world [4] and sequences derived from ancient American samples [8], we obtained a comprehensive picture of the mtDNA gene pools in America, Europe and East Asia. Based on this, we identified the sharing of haplotypes and haplogroups among these regions and between ancient and modern samples. Through this analysis, we traced the maternal ancestry of the modern New World dog populations, assessing for the first time the extent to which American dog breeds descend from the original pre-Columbian population, and reanalysing the ancestry of free-ranging populations in greater phylogeographical detail.

## 2. Material and methods

### (a) Samples, PCR amplification and sequencing

A sample of American dogs, of supposedly indigenous breeds as well as street dogs from both North and South America, was analysed by comparison with a comprehensive sample of European and East Asian dogs (see the electronic supplementary material, table S1). The Old World samples (total, *n* = 1872) were from Europe (*n* = 639), East Asia excluding Siberia (*n* = 889), Siberia (*n* = 95), Africa (*n* = 57), southwest Asia (*n* = 133) and India (*n* = 59). The New World samples (total, *n* = 347) included the Arctic breeds Alaskan Malamute (*n* = 9), Canadian Eskimo dog (*n* = 9), Greenland dog (*n* = 11) and Inuit sled dog (*n* = 18), the North American breeds Chihuahua (*n* = 14) and xoloitzcuintli (*n* = 43), and the South American breed perro sín pelo (Peru, *n* = 53). The American breeds were sampled from pure-bred dogs avoiding known common female ancestry among individuals. Sampling was obtained from European as well as American lineages of the breeds, and for the perro sín pelo, 19 samples were obtained directly from the Peruvian stock. Two American breeds of known Old World origin were also studied: fox Paulistinha (Brazil, *n* = 16) and dogo Argentino (Argentina, *n* = 40). In addition, free-ranging dogs were studied: from North America (USA), the pariah-like Carolina dog [20] (*n* = 19; captive dogs descending from free-ranging dogs); from South America, dogs collected at rural locations (total, *n* = 108); Argentina (Cordova and Buenos Aires regions, *n* = 54; Brazil (Rio de Janeiro and Rio Grande do Norte regions), *n* = 19; Colombia (Bucaramanga region), *n* = 35).

**Figure 1.**
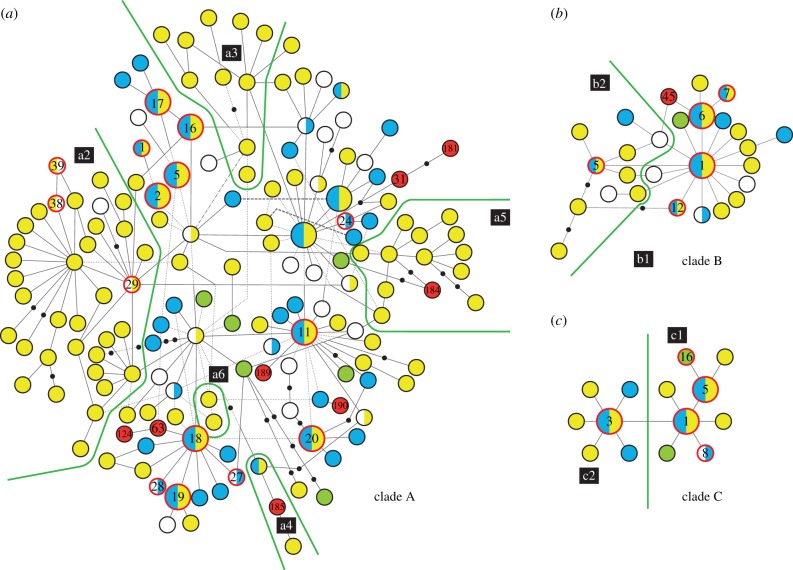
Minimum-spanning network of dog mtDNA control region (582 bp) haplotypes for phylogenetic clades A, B and C. Haplotypes (circles) and empty nodes (solid dots) are separated by one substitutional step (indels are ignored). Phylogenetic subclades were defined based on sequences of mt genomes [4] and are indicated by green lines. Haplotypes found among American dogs are indicated by red colour, haplotypes unique to America by solid red and haplotypes shared with other regions by red outline, and their haplotype names are indicated. Representation of the haplotypes in other regions is indicated as follows. By solid circles: yellow, unique to East Asia (including Siberia) not counting America; green, unique to Siberia not counting America; blue, unique to Europe not counting America. By half circles (indicating sharing of haplotypes): yellow/blue, present in both East Asia and Europe; yellow/white, present in East Asia but absent in Europe; blue/white, present in Europe but absent in East Asia. Solid white, all other geographical representations. Larger sized circles indicate the 15 universal haplotypes (UTs).

The samples were collected in the form of buccal cells or total blood. Buccal cells were collected by swabbing the inside of the cheek using sponge swabs, and applied to Whatman FTA cards (Whatman International Ltd., Maidstone, UK), according to the manufacturer's instructions. Total blood was also sampled on Whatman FTA cards. DNA extractions, PCR amplifications and sequence analysis were performed as previously described [2]. A 582 base-pair fragment of the mt control region (CR) was surveyed using previously published and newly sequenced samples (see the electronic supplementary material, table S1). All novel haplotypes identified in this study have been deposited in GenBank under accession numbers HQ452424–HQ452430 and HQ452435–HQ452438. In a previous study [4], complete mt genomes of 169 individuals representing almost all parts of the CR networks resulted in the identification of subclades within the original clades A, B and C, which are informative since some of them have not been found in European dogs (figure 1). Individuals sequenced only for the CR can be assigned to subclades by mutations within the CR which are diagnostic for each subclade [4].

We also compared our dataset with published data from ancient American dog samples [8] and modern American non-breed dogs [10]. Since the sequences of these two datasets overlapped only partly with our sequences, comparisons were performed separately from the main analysis. Thus, our modern sequences were compared with 19 ancient DNA sequences obtained from dog remains from South America (Bolivia, Peru and Mexico) and Alaska (Fairbanks area) [8]. These sequences overlap by 244 bp with our modern sequence data. We also compared our samples with sequences from Castroviejo-Fisher *et al*. [10] which consisted of mtDNA sequences from ‘400 dogs from rural and isolated areas as well as street dogs’ from all across America. These sequence overlap by 342 bp with our sequences.

### (b) Phylogenetic analysis

Sequences were aligned with the MUSCLE tool implemented in Geneious v. 5.5.3 Pro [21]. Comparisons of sequences and identification of haplotypes were performed with DnaSp v. 5 [22]. Minimum-spanning networks and trees were constructed manually based on the minimum distances between haplotypes calculated with Arlequin v. 3.5 software [23] ignoring indels. Unique and shared haplotypes among dog populations or breeds from different geographical regions were identified using the program UPST.EXE (available by request to zhangab2008@mail.cnu.edu.cn).

## 3. Results

Among the American samples, all haplotypes belonged to the universally occurring clades A, B and C, indicating an Old World origin for all maternal lineages. Among the Old World samples, 38 haplotypes were unique to Europe, 121 unique to East Asia (excluding Siberia) and 12 unique to Siberia (figure 1). This dataset included 302 new European samples relative to a previous study [4], but the large difference between European and East Asian samples observed previously did not alter. Thus, the diversity among European dogs was very limited, with practically all haplotypes centred around the 15 UTs (figure 1). By contrast, approximately 50% of East Asian dogs carried haplotypes on large distance from those found in Europe, many of which belonging to sub-haplogroups not detected in Europe, implying the possibility of tracing the geographical origins of a large proportion of the American maternal lineages.

### (a) Breed dogs

#### (i) Arctic America

Inuit sled dogs, Canadian Eskimo dogs and Greenland dogs had similar mtDNA gene pools, in that the most frequent haplotype for all three breeds was A31, unique to this group of breeds (table 1 and figure 1). The Inuit sled dog and the Greenland dog also shared a haplotype unique to the two breeds (A124). In addition, the Inuit sled dog and the Eskimo dog had one unique haplotype each (A181 and A63, respectively). Three of these four private haplotypes differ by at least two substitutions from any of the European haplotypes. In total, 30/38 individuals in this group of dogs had haplotypes unique to the group, and the remaining eight dogs had universally occurring haplotypes, present in both Europe and East Asia. Importantly, a very recent article [9] showed that pre-Columbian samples from Greenland had a high frequency of A31, strongly suggesting ancestry from the pre-Columbian population for this group of dogs.

**Table 1.** mtDNA control region haplotypes found among American breed dogs and Carolina dog, and their geographical distribution in both the Old World and the New World. Subclade refers to the 10 subclades of clades A, B and C; only haplotypes belonging to subclades other than the four universal subclades a1, b1, c1 and c2 are indicated. UT, universal types of DNA haplotype found worldwide [4]; PT, private types found only in a specific breed, population and/or region.

breed (code)	*n*	haplotype (subclade)	frequency (*n*)	geographical distribution
Inuit sled dog (ISD)	18	A181	6% (1)	PT
		A124	6% (1)	PT in Arctic America (ISD, GD)
		A31	78% (14)	PT in Arctic America (ED, GD, ISD)
		A20	11% (2)	UT
Eskimo dog (ED)	9	A31	67% (6)	PT in Arctic America (ED, GD, ISD)
		A63	11% (1)	PT
		A18	22% (2)	UT
Greenland dog (GD)	11	A31	55% (6)	PT in Arctic America (ED, GD, ISD)
		A124	9% (1)	PT in Arctic America (ISD, GD)
		A11; A17; A20	36% (4)	UT
Alaskan Malamute (AM)	9	A29 (a2)	78% (7)	Siberia, Japan, China, Indonesia
		A11; A17	22% (2)	UT
Chihuahua (CH)	14	A185 (a4)	36% (5)	PT
		C16	7% (1)	South America (CH and PSP), Siberia
		A11; A17; B1	57% (8)	UT
xoloitzcuintle (Xo)	43	A24	2% (1)	Europe, Colombia
		B12	2% (1)	Europe, Japan
		A2; A16; A17; B1; C1; C2; C3	95% (41)	UT
Carolina dog (CD)	19	A184 (a5)	37% (7)	PT
		A39 (a2)	5% (1)	Japan, China
		A16; A18; A19; B1	58% (11)	UT
perro sin pelo Del Peru (PSP)	53	C16	62% (33)	Siberia, South America (CH, PSP)
		A1	2% (1)	Europe , China, Korea, Brazil
		B8	2% (1)	Europe
		C8	2% (1)	Europe
		A2; A11; A17; A18; A19; B1	32% (17)	UT

**Figure 2.**
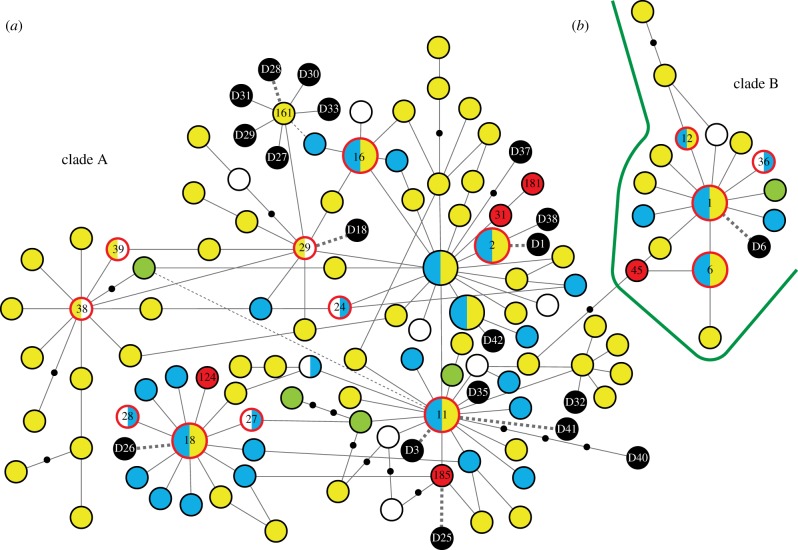
Minimum-spanning network of dog mtDNA control region (244 bp) haplotypes for clades (A and B), comparing the modern samples with ancient American sequences from Leonard *et al.* [8]. Haplotypes (circles) and empty nodes (solid dots) are separated by one substitutional step (indels are ignored). The colours of the circles represent the geographical distribution of haplotypes as described in the legend to figure 1. Black solid circles indicate the ancient sequences, the numbering indicating the haplotype names used in Leonard *et al*. Dashed lines indicate that the ancient sequence is identical to the connected modern haplotype. The haplotypes of the modern samples sometimes correspond to several different full-length haplotypes (based on 582 bp); the name of one among these is indicated for haplotypes found among American samples. Larger sized circles represent universal haplotypes (UTs).

Among the Alaskan Malamute, 7/9 individuals had haplotype A29, which belongs to subclade a2 (not represented in Europe) and otherwise found only in East Asia (including Siberia) and island Southeast Asia. The two other individuals had universally occurring haplotypes. Haplotype A29 was also found in pre-Columbian samples from Alaska [8,9]. This suggests ancestry of modern Alaskan Malamute from the pre-European Alaskan population (figure 2) but, since A29 is also commonly found among Siberian husky with which Alaskan huskys were interbred in the early 1900s, the evidence for this is not clear cut. The Alaskan Malamute and the Eskimo/Greenland/Inuit group had almost totally different mtDNA gene pools, only 2/9 and 2/38 individuals, respectively, sharing haplotypes between the groups.

#### (ii) North America

For the Mexican breed Chihuahua, the most frequent haplotype (carried by 5/14 individuals) was A185, which was unique to this breed among the modern dogs. Importantly, this haplotype was also found in one pre-Columbian sample from Mexico (figure 2), suggesting direct ancestry of Chihuahua from ancient Mexican dogs. One individual had haplotype C16, the most common type among the South American breed perro sín pelo del Peru. C16 was also reported for xolo at a frequency of 16% in a previous study [24]. Among the Old World samples, C16 was found only among Siberian dogs. The remaining Chihuahua had universally occurring haplotypes. The Mexican ‘naked breed’ xoloitzcuintle (xolo) had only haplotypes occurring universally and two haplotypes found in Europe. However, as noted above, C16 was found in a previous study of xolo, at a frequency of 16% [24]. Notably, 79% of the xolo carried haplotypes shared with perro sín pelo del Peru, the South American ‘naked breed’.

#### (iii) South America

For the Peruvian ‘naked breed’ perro sín pelo del Peru, the majority of dogs (33/53) had haplotype C16, absent among the European samples and shared only with the other ‘naked breed’ xolo, and with Chihuahua and Siberian dogs. Two individuals had haplotypes otherwise unique to Europe, and the remaining individuals had universally occurring haplotypes. Eighty-one per cent of the individuals shared a haplotype with xolo.

In conclusion, all investigated breeds of presumed indigenous origin across America carried haplotypes indicating pre-European origin, except for Alaskan Malamute for which the results were ambigous. All these breeds except xolo had a high frequency of haplotypes absent in Europe: 79% of the Arctic Inuit, Eskimo and Greenland dogs, and a total of 35% of dogs belonging to the Meso/South American breeds Chihuahua, xolo and perro sín pelo del Peru. Only half of the lineages introduced to America from Asia are expected to differ from European sequences, since the frequency of UTs is approximately 50% in Siberia and East Asia. Therefore, even if 100% of the lineages would have an ancient origin from Asia, only 50% of the individuals would be expected to carry haplotypes absent in Europe. Hence, our data indicates that the Arctic breeds Inuit, Eskimo and Greenland dog (having a frequency above 50% of haplotypes absent in Europe) have remained practically uninfluenced by European lineages, and that among the Meso/South American breeds (35% of the dogs carrying haplotypes distinct from European haplotypes), 70% of the maternal lineages originate from the pre-Columbian population. Thus, replacement of indigenous American breeds by European dogs seems to have been relatively limited.

We also analysed dogs belonging to the fox Paulistinha and dogo Argentino breeds. These breeds originate from dogs of known European ancestry. In accordance, these dogs carried only haplotypes that are frequently found in Europe, five universal haplotypes and one other haplotype frequent in Europe (see the electronic supplementary material, table S2).

### (b) Comparison of pre-Columbian sequences with a comprehensive modern sample

We compared our dataset with ancient American mtDNA sequences from Mexico, Peru, Bolivia and Alaska reported in an earlier study ([8]; figure 2). This analysis is the first comparison of these ancient American sequences with a comprehensive collection of sequences from modern East Asian dogs and American breed dogs, instead of predominantly European breeds as in earlier studies. This allowed us to investigate possible geographical continuity over time among American breed dogs and to explore the possibility of an East Asian origin of the ancient American dog mtDNA lineages.

We found two cases where ancient and modern samples from the same geographical region had the same non-universal haplotype, suggesting continuity since pre-European time for these populations. In one case, haplotype A185 was found exclusively in ancient Mexican samples (D25) and in modern samples of the Mexican Chihuahua, strongly indicating direct ancestry of Chihuahua from Mexican pre-Columbian dogs. Furthermore, a haplotype corresponding to A29 (D18), otherwise found only in Siberia, East Asia and Oceania, was identified in ancient Alaskan samples as well as in the modern Arctic breed Alaskan Malamute but, as related above, the presence of A29 also among Siberian husky offers an alternative to genetic continuity in Alaska. Notably, a very recent study of pre-Columbian dogs from Alaska and Greenland [9] showed that, in Greenland, both the ancient population and the modern Inuit sled dog population carry predominantly the unique haplotype A31, strongly indicating ancient local ancestry for this group of Arctic dogs.

Importantly, Leonard *et al*. reported one phylogenetic group of haplotypes (containing haplotypes D27, D28, D29, D30, D31 and D33) to be unique for the ancient South/Meso American samples (figure 2). However, we found the central haplotype D28 in East Asia (in Korea, haplotype A161), thus placing the origins of these sequences in East Asia. Eight of the 19 ancient haplotypes correspond to haplotypes found in the modern samples, all of which, except the American specific A185, found in East Asia. The remaining haplotypes were unique to the ancient samples, but differ by one or two substitutions from extant haplotypes found in East Asia, except one ancient haplotype (D40). D40 is separated by at least four substitutions from any other dog haplotype and seemingly represents a separate phylogenetic clade (figure 2). Therefore, D40 is possibly the result of a crossbreeding between a dog and a female wolf in America.

In conclusion, we find a strong indication that the Mexican breed Chihuahua has a direct ancestry from Mexican pre-Columbian dogs, and a similar link was recently shown for Inuit dogs and pre-Columbian samples in Greenland [9]. Importantly, except D40, all ancient American samples can be linked to haplotypes found among East Asian dogs.

### (c) Free-ranging dogs

Among the ‘free-ranging’ Carolina dogs of southern USA, the most frequent haplotype was A184, which was unique to these dogs (it is a novel halpotype not previously reported in GenBank) and belongs to the East Asian-specific phylogenetic subclade a5 (figure 1). One individual had haplotype A39, otherwise found only among Chinese non-breed dogs and the Japanese breed shiba inu. The remaining dogs had universal haplotypes. This gives a strong support to the hypothesis that the Carolina dog has indeed originated from pre-Columbian dogs.

By contrast, South American free-ranging dogs from Argentina, Brazil and Colombia had mainly universally occurring haplotypes (83.3% carrying UTs) and several European specific haplotypes (see the electronic supplementary material, table S2). There were also three unique haplotypes (A189, A190, B45), a single step from haplotypes found also in Europe, and three haplotypes (A38, B2 and B5) absent from Europe but present in East and West Asian breeds (e.g. chow chow, Afghan hound and akita) and rural non-breed dogs. Thus, South American free-ranging dogs in our sample seem to originate mainly from European dogs, although traces of native dogs cannot be totally excluded.

This is in good agreement with a previous study by Castroviejo-Fisher *et al*. [10] of 400 dogs from rural and isolated areas, as well as street dogs from across America. This study reported that a very low proportion of the mtDNA lineages among these modern American non-breed dogs (less than 10%) derive from the pre-European dog population, based on a comparison with the 19 ancient American dog samples reported by Leonard *et al.* [8]. This indicated that almost all indigenous dogs were replaced by European dogs. However, comparing the dataset in Castroviejo-Fisher *et al*. with our comprehensive sample of modern dogs, we found that these dogs carry several potentially indigenous mtDNA haplotypes which are unique to America or otherwise found exclusively in East Asia (see the electronic supplementary material, figure S1 and table S3). These haplotypes were carried by 7.8% of all dogs, which indicates that approximately 15% of the lineages have pre-European ancestry, considering that at least 50% of indigenous American dogs are expected to carry non-informative haplotypes (UTs) also found in Europe. More importantly, in a detailed analysis of the different subpopulations, we also noted that the putatively indigenous haplotypes were not evenly distributed. Thus, 9.8% of the Mexican dogs and 14.8% of the Bolivian dogs carry these haplotypes (indicating 20–30% Asian ancestry), but only 2.3% of the dogs from Argentina. This agrees with our findings that a large proportion of Carolina dogs carry seemingly indigenous American haplotypes, whereas our sample of free-ranging Argentinan dogs has almost exclusively European haplotypes. Therefore, it seems that while free-ranging American dogs generally derive from European dogs, there are pockets of rural populations, for example, the Carolina dog and populations in Mexico and Bolivia, with a high proportion of pre-Columbian ancestry. Importantly, comparing with a dataset from Europe [25], only two of 94 dogs of European origin (2.1%) carried a unique haplotype, confirming that the majority of the unique American haplotypes have an indigenous origin and are not merely European haplotypes not yet sampled.

Castroviejo-Fisher *et al.* [10] also reported that a large proportion of haplotypes found in the dataset (23 out of 40) were unique to the American dogs compared with dogs of the Old world. However, the comparison was performed against a limited dataset of non-American dogs [26], and comparing instead to our more comprehensive dataset of dogs from across the Old World, we identified the majority of these 23 haplotypes. For the 342 bp region overlapping with our dataset, there were 34 haplotypes in the dataset of Castroviejo-Fisher *et al*. Of these, 27 were shared with dogs in Eurasia, leaving only seven haplotypes unique to America.

## 4. Discussion

This study provides clear evidence for the ancient Asian ancestry of extant American dog breeds. All breeds of presumed indigenous American origin carried haplotypes absent in a comprehensive sample of European dogs, and our data indicates that only 30% or less of the female lineages in indigenous American breeds have a European origin. Importantly, the Mexican breed Chihuahua shared a haplotype uniquely with Mexican pre-Columbian samples, showing genetic continuity over time and geographical region and corroborating the Mexican origins of the Chihuahua. The data also once more confirmed that American dogs have a common origin with Old World dogs, since no distinct haplogroups unique to American dogs were found and all haplotypes fell into the previously described universal phylogenetic clades A, B and C. However, we note that one pre-European Alaskan dog reported by Leonard *et al*. [8] had a haplotype (D40) separated by four substitutions from the closest modern dog haplotype, which we believe may derive from a dog–wolf hybridization.

An important finding is that not only American breed dogs but also some populations of free-ranging dogs seem to stem from indigenous American dogs. Thus, we here give genetic evidence that feral dogs from the USA, the so-called Carolina dog, may have an indigenous American origin and are not just ‘run-away’ dogs of European descent. The dingo-like appearance of the Carolina dogs may therefore be a remnant of their ancient past. The most frequent haplotype (A184) is unique to Carolina dogs and belongs to an East Asian-specific phylogenetic subclade, offering a clear indication of an East Asian origin for these dogs. Also haplotype A39, otherwise found only among East Asian dogs including the shiba inu, may have an ancient East Asian origin, but the possibility remains that this haplotype derives from abandoned shiba. The South American free-ranging dogs carried mainly universal or typically European haplotypes, but a few individuals had haplotypes carried by East Asian dogs and absent among European breeds. However, all these haplotypes are also carried by East Asian breeds present in the Western world dog population and could have resulted from integration of abandoned Asian breed dogs. We conclude that the South American free-ranging dogs mainly originate from dogs brought by the Europeans, although traces of native dog cannot be totally excluded.

In general agreement with our data, the study by Castroviejo-Fisher *et al.* [10] reported that at most 10% of the mtDNA lineages among American rural and street dogs have indigenous American origin. However, in a more detailed phylogeographical analysis of these samples, we note that in the subsamples from Mexico and Bolivia approximately 25% of the lineages seem to retain mtDNA haplotypes with an Asian origin. Therefore, pockets of remaining populations with high proportion of indigenous pre-European origin seem to persist among American non-breed dogs, as well as among the indigenous American breeds.

We conclude that the ancestry of the American dog population is complex. There are several different types of populations of American dogs of possibly pre-Columbian origin, and these populations retain different proportions of pre-Columbian ancestry. The dog breeds of supposedly indigenous American origin carry predominantly mtDNA haplotypes of pre-Columbian origin. By contrast, free-ranging dogs, including dogs from rural areas as well as street dogs, generally show no or very little pre-Columbian ancestry, but also among these dogs there remain populations with considerable proportions of indigenous American ancestry.

The studied Arctic American dogs seem to consist of two separate populations. The Alaskan Malamute is thought to descend from dogs bred by the Mahlemut people of the upper-western Alaska. A majority of these dogs carried haplotype A29, which seemingly presents both spacial and temporal clues to the origins of the breed. A29 (absent in Europe) is found among East Asian dogs and, significantly, among Siberian husky, a sled dog originally bred by the Chukchi people on the northeastern tip of Siberia. This indicates genetic links between East Asia, Siberia and Arctic America. A29 was also found in ancient samples from Alaska, indicating the presence of the Alaskan Malamute ancestors in the pre-Columbian era. However, a complicating factor is that Alaskan Malamutes were interbred with Siberian husky in the gold rush era; analyses of additional markers will be necessary to establish how the modern Alaskan Malamutes relates to the dogs of the Mahlemut people. The other modern Arctic breeds (Inuit, Eskimo and Greenland dogs) seem to constitute a separate genetic group, as only 2/38 individuals shared haplotypes with Alaskan Malamutes. The three breeds share a common gene pool including a unique haplotype (A31) found in the majority of individuals. These three breeds, in accordance with the mtDNA data, are often considered a single breed and are thought to descend from sled dogs bred by Thule people, the ancestors of the modern Inuits. This culture developed in coastal Alaska by 1000 years ago and rapidly reached Greenland a few centuries later, having presented the first evidence of consistent use of sled dogs in the Arctic [27]. Importantly, a very recent study of pre-Columbian arctic dogs [9] showed that the related ancient dog population in Greenland carried almost exclusively A31. Thus, the ancient and modern populations share the unique haplotype A31 at high frequency, confirming the ancestry of modern Inuit, Eskimo and Greenland dogs from the local ancient population. The almost total lack of shared haplotypes between these breeds and the Alaskan Malamute possibly reflects sequential arrival to America of the related human populations [15].

Overlap was also minimal between the Arctic breeds and other dogs across America. By contrast, North American (excluding the Arctic) and South American breeds showed genetic links. Chihuahua shared one haplotype (C16) exclusively with perro sín pelo and Siberian dogs. This haplotype was absent from our xolo sample but was found in this breed in a previous study [24]. Furthermore, around 80% of the individuals of the Mexican ‘naked breed’ xolo and the Peruvian ‘naked dogs’ shared haplotypes, indicating a geographical link connected with the shared morphology, in agreement with all naked breeds carrying the same causative mutation [28]. It is notable that genetic data for both dogs and humans indicate an initial migration forming the North and South American populations, followed by subsequent waves into the Arctics [15].

Comparison of the modern data with ancient sequences suggested genetic continuity from pre-Columbian into modern times in Mexico and, possibly, Alaska, adding to the evidence for genetic continuity in Greenland presented very recently by Brown *et al.* [9]. Modern Chihuahua and ancient Mexico had the same unique haplotype (A185 and D25, respectively), and ancient Alaska and modern Alaskan Malamute shared a non-European haplotype (D18 and A29, respectively). Interestingly, A185 and A29 have been reported also for modern Puerto Rican ‘street dogs’ [29], but the possible link to ancient American samples was not noted in that study. This suggests that A185 and A29 may have been widespread across ancient America but, since the two haplotypes are found among modern Chihuahua and Siberian husky, there remains the possibility that A185 and A29 in Puerto Rico originate from abandoned dogs of these breeds. An important finding when comparing the new modern sequences presented in this study with the ancient DNA samples was that the central haplotype (D28) in a formerly ‘ancient America-specific’ clade [8,10] was found among East Asian dogs (haplotype A161). This implies that all ancient American sequences except one (D40; possibly the result of dog–wolf crossbreeding) can now be linked to haplotypes present in East Asia or Siberia. We also note a large proportion of unique haplotypes among the ancient samples. However, the ancient sequences harbour an unusual amount of transversions, suggesting that these may be single base substitution artefacts, a known problem in the sequencing of ancient samples. For example, the five unique haplotypes surrounding D28 (D27, D29, D30, D31 and D33) are defined by three transversions (in previously non-variable positions) and two transitions, compared with six transversions and 50 transitions found among the 1555 modern samples in clade A. It is therefore possible that several of the haplotypes surrounding D28 are in reality D28 (modern A161), and thus identical to modern East Asian samples.

In the modern sample, we identified nine haplotypes, five in the Arctic and four in North and South America, which were distinct from haplotypes found among European dogs. Only 50% of the pre-European dogs are expected to have carried haplotypes absent in Europe, implying that several more of the American lineages probably have an Asian origin. There were also at least four additional haplotypes found in the ancient American samples. Thus, it seems that the American dog population was not formed through a severe genetic bottleneck, but that several lineages were brought to America from Asia in the pre-Columbian era, probably in several waves of migration. The analyses in this study are based on mtDNA, which is a single genetic marker inherited maternally, putting obvious limitations to the inferences. To assess fully the pre- and post-Columbian contribution of genetic material to the American breeds, as well as the possibility of hybridization between American dogs and wolves, studies based on additional markers, suggestively the Y-chromosome as well as autosomal markers will be necessary. Possibly, improved phylogeographic fine mapping of both American humans and dogs may show whether the genetic diversity of domestic dogs mirrors that of humans and provide clues for understanding the colonization of the New World.
